# Exploring the psychological and physiological effects of a virtual reality-based bicycle exercise in hemodialysis patients: a randomized controlled trial

**DOI:** 10.1038/s41598-026-42972-4

**Published:** 2026-05-12

**Authors:** Jing Hu, Zheng Gong, Jingjing Huang, Juanjuan He, Xiaoli Zhu, Lixia Yan, Haoyue Wang

**Affiliations:** 1 Department of Cardiology, The No. 904th Hospital of Joint Logistic Support Force, Changzhou, 213000 Jiangsu China; 2 Anesthesiology Department, The No. 904th Hospital of Joint Logistic Support Force, Changzhou, 213000 Jiangsu China; 3 Political Office, The No. 904th Hospital of Joint Logistic Support Force, Changzhou, 213000 Jiangsu China; 4 Research Center of Mental Disease, The No. 904th Hospital of Joint Logistic Support Force, Changzhou, 213000 Jiangsu China; 5 Hemodialysis Department, The No. 904th Hospital of Joint Logistic Support Force, Changzhou, 213000 Jiangsu China; 6 Nursing Department, The No. 904th Hospital of Joint Logistic Support Force, Changzhou, 213000 Jiangsu China

**Keywords:** VR-based bicycle exercise, Depression, Anxiety, Hemodialysis nursing, Biomarkers, Diseases, Health care, Nephrology

## Abstract

This study aimed to evaluate the efficacy of a virtual reality (VR)-based bicycle exercise in psychological distress (depression and anxiety) and selected biochemical parameters among patients receiving maintenance hemodialysis (MHD). A total of 70 patients from the dialysis station at Changzhou Medical District of the No. 904th Hospital were randomly allocated into study group(VR-based bicycle exercise, n = 35) and control group(routine nursing care, n = 35). The depression and anxiety levels were assessed by Self-rating Depression Scale (SDS), and Self-rating Anxiety Scale (SAS) respectively. Physiological indicators, including serum creatinine (Scr) and blood urea nitrogen (BUN) levels, were also analyzed. Following the intervention, the study group exhibited significant reductions in SDS and SAS scores (both *P* < 0.05). The levels of Scr and BUN in peripheral blood were also observed to be significantly decreased in study group (*P* < 0.05). Correlation analysis revealed a significant positive correlation between SAS/SDS scores with both Scr and BUN levels (*P* < 0.05 or *P* < 0.01). Furthermore, regression models identified SAS scores as significant predictors of Scr and BUN levels, accounting for 27.8% and 31.9% of their variance, respectively (*P* < 0.05 or *P* < 0.01). VR-based bicycle exercise can improve psychological well-being, and was associated with beneficial changes in pre-dialysis serum biomarkers (Scr and BUN) in MHD patients. This integrated intervention represents a promising non-pharmacological strategy to complement standard care in MHD patients.

## Introduction

Maintenance hemodialysis (MHD) served as the primary renal replacement therapy for patients with end-stage renal disease (ESRD), represents a widely utilized, technically feasible, and safe blood purification modality^1^. However, Chronic kidney disease (CKD) and long-term dialysis make these patients negative emotional states, including depression and anxiety. Evidence indicates that such psychological distress may disrupt phenylalanine metabolism in MHD patients, resulting in increased blood urea nitrogen (BUN) and serum creatinine (Scr) levels, thereby potentially exacerbating disease progression^2^. Current clinical approaches primarily incorporate pharmacological therapy, psychological support and physical exercise to reduce the symptom burden and emotional disturbances. Nevertheless, in MHD patients, pharmacologic management of symptoms and mood disorders may aggravate mineral metabolism abnormalities and increase infection risks. Therefore, therapeutic decisions should be based on a comprehensive consideration of drug metabolism profiles, dialysis-related factors, and individual psychological needs. Given the substantial healthcare and caregiver burdens associated with long-term dialysis, non-pharmacological interventions are increasingly regarded as a preferable strategy in clinical practice.

Studies have demonstrated that moderate physical activity can enhance physical fitness, alleviate fatigue and weakness, reduce muscle fatigue and pain associated with dialysis, and improve negative emotional states in MHD patients. Aerobic exercises, particularly stationary cycling, have been shown to effectively improve exercise capacity and circulatory function^3–5^. However, exercise adherence remains suboptimal, and the implementation of cycling interventions in routine clinical practice presents significant challenges. Virtual Reality (VR) technology is defined as a system that creates simulated three-dimensional environments through the delivery of multi-sensory, immersive, interactive, and motivational stimuli via integrated audiovisual interfaces^6,7^. Owing to its engaging nature, safety, ease of use, and scalability, VR has been increasingly adopted in clinical settings to improve patient treatment compliance^8^. This study aims to evaluate the effect of a VR-based bicycle exercise from psychological and physiological perspectives on MHD patients.

## Materials and methods

### Subjects

#### Participant flow and adherence

A total of 90 maintenance hemodialysis patients were assessed for eligibility between September 2022 and September 2024 from the Changzhou Medical Area of Hospital No. 904. Of these, 14 were excluded: 10 did not meet the inclusion criteria, and 4 declined to participate. The remaining 76 eligible participants provided informed consent and were randomized.

Using a computer-generated random sequence with sealed opaque envelopes, participants were allocated in a 1:1 ratio to either the control group (n = 38) or study group (n = 38).

During the 12-week intervention period, 6 participants (7.9%) discontinued the study, with an equal number of dropouts in each group (n = 3 per group). In the VR-cycling group, reasons for discontinuation were: kidney transplantation (n = 1) and withdrawal due to personal reasons (n = 2). In the control group, reasons were: transfer to another dialysis unit (n = 2) and hospitalization for an unrelated medical event (n = 1).

Therefore, 70 participants (92.1% of those randomized) completed the entire study protocol, with 35 participants in each group available for the final assessment. The primary analysis was conducted on this per-protocol population. Adherence to the intervention in the VR-cycling group was high, with participants completing an average of 33.2 out of 36 scheduled sessions (mean adherence rate: 92.5%).

#### Inclusion an exclusion criteria

Inclusion Criteria: (1) undergoing maintenance hemodialysis for ≥ 3 months with a frequency of three sessions per week; (2) absence of psychiatric or neurological disorders known to cause cognitive impairment (e.g., Alzheimer’s disease or chronic psychosis); (3) medically stable with normal cardiac function, lower limb muscle strength of grade ≥ 3, and good motor function; (4) voluntary participation with signed informed consent. Exclusion Criteria: (1) contraindications to physical activity; (2) use of medications affecting balance within the preceding 6 months; (3) inability to cooperate with or complete the exercise protocol; (4) history of fracture within the preceding 6 months; (5) use of a femoral vein catheter for vascular access.

#### Baseline characteristics of the study participants

The control group comprised 21 males and 14 females, aged 18–55 years (mean ± SD: 41.49 ± 6.65). Marital status was distributed as follows: 28 married and 7 others. The study group consisted of 19 males and 16 females, aged 18–55 years (mean ± SD: 41.63 ± 6.74), with 30 married and 5 others. No statistically significant differences were observed in the baseline demographic characteristics between the two groups (*P* > 0.05). This study was approved by the Clinical Research Ethics Committee of the No. 904th Hospital of Joint Logistic Support Force (Approval No. 2022-08-001). All procedures involving human participants were performed in accordance with the ethical standards of the institutional and/or national research committee and with the 1964 Helsinki Declaration and its later amendments or comparable ethical standards. Written informed consent was obtained from all individual participants prior to enrollment. The interventions in this study consisted of routine clinical care procedures that are standard in hemodialysis management, with no additional risks or invasive procedures beyond those encountered in daily medical practice. The study was conducted in full compliance with relevant ethical guidelines and regulatory requirements.

## Methods

### Study design and procedures

All patients underwent hemodialysis following a standard protocol, with close monitoring during dialysis. Participants in the control group received only the standard, without any additional structured exercise intervention. Study group received the identical standard, routine pre-dialysis care plus the adjunctive VR-cycling exercise intervention. Adverse events such as hypotension or hypoglycemia were promptly managed. Patients were instructed to monitor and record parameters including blood pressure, blood glucose, and body weight during dialysis. Pre-dialysis interventions included health education, medication guidance, fluid management, vascular access care, dietary guidance, activity recommendations, lifestyle advice, and sleep interventions.

#### VR-cycling intervention details

A designated nurse provided bedside education on hemodialysis procedures and related precautions. (1) Team Establishment: A team consisting of one rehabilitation therapist and three nurses was formed. The rehabilitation therapist was responsible for training the nurses in the VR cycling protocol and providing exercise guidance to patients. (2) VR Equipment: The Sonic VR all-in-one device was used. The VR equipment for nursing staff primarily included a head-mounted device (weight: 462 g) and controllers (weight: 80 g each). The exercise sessions were scheduled during the first two hours of dialysis, three times per week, with each session lasting 30 min. Prior to exercise, patients were connected to electrocardiographic monitoring to ensure safety. The Borg Rating of Perceived Exertion (RPE) scale was administered every 5 min. Each exercise session consisted of three phases: a warm-up (5 min of lower-limb stretching and low-intensity aerobic exercise, RPE 8–9, corresponding to “very light”), a training phase (20 min of cycling exercise, RPE 11–13, corresponding to “fairly light” to “somewhat hard”), and a cool-down phase (5 min of low-intensity, non-load-bearing activity, RPE 8–9). During the initial intervention period, exercise duration was carefully controlled, and patient responses were closely observed. Patients reporting excessive fatigue were allowed to rest within a designated area. Any participant experiencing VR-related discomfort was advised to reduce or pause their movement and inform the staff^9–11^. The intervention lasted for 12 weeks, all outcome measures were assessed at Baseline (Week 0) and immediately post-intervention (Week 13).

### Observation indicators

#### Psychological scales (SDS/SAS)

For the enrolled hemodialysis patients, the Chinese versions of Self-Rating Depression Scale (SDS) and Self-Rating Anxiety Scale (SAS) were used respectively, which have been validated in chronic disease populations. The SDS contains 20 items, each item is scored on a 4-point Likert scale from 1 to 4. The total raw score ranges from 20 to 80. The standard method involves calculating an Index Score: Index Score = (Total Raw Score/80) × 100. SAS also contains 20 items, scored on a 4-point Likert scale from 1 to 4. The total raw score ranges from 20 to 80. Similarly, an Index Score is calculated: Index Score = (Total Raw Score/80) × 100. The higher the score, the more severe the depression and anxiety.

#### Pre-dialysis biochemical parameters (Scr/BUN levels)

Venous blood samples were collected pre-dialysis on the mid-week session. Scr and BUN levels were determined using standardized enzymatic methods with commercial assay kits from [Roche Diagnostics] on an [Cobas c501] automated biochemical analyzer. All assays were performed according to the manufacturer’s protocols, with internal quality control procedures followed routinely.

### Statistical analysis

Statistics were analyzed using SPSS 22.0, categorical data are presented as numbers and percentages (%) and were analyzed using the chi-square (χ^2^) test. Normally distributed continuous data are expressed as the mean (± standard deviation). Between-group differences at post-intervention were analyzed using Analysis of Covariance (ANCOVA), with the post-intervention score as the dependent variable, group as the fixed factor, and the baseline score as a covariate. Effect sizes for between-group differences are reported as Cohen’s d, interpreted as small (0.20), medium (0.50), and large (0.80). Adjusted mean differences with 95% Confidence Intervals are presented. Relationships among variables were assessed using Pearson correlation and linear regression analyses. *P* < 0.05 was considered statistically significant.

## Results

### Comparing of the scoring of psychological scales between study group and control group before and after the VR-based bicycle exercise

No significant differences in depression and anxiety scores were observed between the two groups at baseline (*P* > 0.05) (Table 1.). Following the intervention, both groups exhibited significant reductions in these scores. However, the reduction of study group was significantly greater that in control group (*P* < 0.05). The ANCOVA revealed SDS score’s adjusted mean difference = − 7.50 points (95% CI: − 10.23 to − 4.77), *P* < 0.001, SDSwith a large effect size (Cohen’s d = 0.95); SDS score’s adjusted mean difference = − 10.50 points (95% CI: − 12.80 to − 8.20), *P* < 0.001, with a large effect size (Cohen’s d = 1.25).

**Table 1:** Comparison of psychological outcomes between groups.

Scale	Group	N	Baseline (Mean ± SD)	Post-intervention (Mean ± SD)	Adj. MD (95% CI)	*P* value	Cohen’s d
SDS	Study	35	48.03 ± 12.35	37.14 ± 8.23	− 7.50 (− 10.23, − 4.77)	< 0.001	0.95
	Control	35	48.23 ± 9.60	45.91 ± 10.75			
SAS	Study	35	46.46 ± 11.49	31.49 ± 5.30	− 10.50 (− 12.80, − 8.20)	< 0.001	1.25
	Control	35	46.60 ± 8.53	42.74 ± 11.49			

Adj. MD = Adjusted mean difference; CI = Confidence interval. The adjusted mean difference, *P* value, and Cohen’s *d* were derived from Analysis of Covariance (ANCOVA), with the baseline score as a covariate. Effect size (Cohen’s *d*) was interpreted as small (0.20), medium (0.50), and large (0.80).

### Comparison of SCr and BUN levels between the study group and control group before and after the VR-based bicycle exercise

After the exercise intervention, the levels of Scr and BUN in both control group and study group showed a downward trend. Moreover, the decrease in study group was significantly greater than that in control group (*P* < 0.05) (Table 2.). The ANCOVA revealed Scr level’s adjusted mean difference = − 35.00 points (95% CI: − 49.20 to − 20.80), *P* < 0.001, SDSwith a large effect size (Cohen’s d = 0.80); BUN level’s adjusted mean difference = − 1.80 points (95% CI: − 2.30 to − 1.30), *P* < 0.001, with a large effect size (Cohen’s d = 1.25).

**Table 2:** Comparison of pre-dialysis biochemical parameters between groups.

Parameter	Group	N	Baseline (Mean ± SD)	Post-Intervention (Mean ± SD)	Adj. MD (95% CI)	*P* value	Cohen’s d
Scr (μmol/L)	Study	35	407.97 ± 11.20	260.14 ± 8.23	− 35.00 (− 49.20, − 20.80)	< 0.001	0.80
	Control	35	407.57 ± 12.45	296.43 ± 13.15			
BUN (mmol/L)	Study	35	12.60 ± 1.45	9.58 ± 1.07	− 1.80 (− 2.30, − 1.30)	< 0.001	1.10
	Control	35	12.62 ± 1.49	11.49 ± 1.25			

Adj. MD = Adjusted mean difference; CI = Confidence interval; Scr = Serum creatinine; BUN = Blood urea nitrogen. All samples were collected pre-dialysis. The adjusted mean difference, *P* value, and Cohen’s d were derived from Analysis of Covariance (ANCOVA), with the baseline score as a covariate. Effect size (Cohen’s d) was interpreted as small (0.20), medium (0.50), and large (0.80).

### Correlation of SDS and SAS Scores with Scr and BUN levels in MHD patients

According to Pearson correlation analysis, the serum Scr and BUN levels were significantly positively correlated with the scoring of SDS/SAS scales (*P* < 0.05 or *P* < 0.01) in MHD patients (Table 3).

**Table 3:** Correlation of depression and anxiety score with Scr and BUN levels in MHD Patients(r).

variable	SDS	SAS
Scr	0.326**	0.446**
BUN	0.207*	0.524**

*P < 0.05, **P < 0.01.

### Multivariate regression analysis of factors associated with the levels of Scr and BUN in MHD patients

Separate multiple regression analyses were performed with Scr/BUN levels as the dependent variables, and SDS/SAS scores as independent variables. The analysis indicated that SAS scores significantly predicted Scr/BUN levels (*P* < 0.05), with the models explaining 27.8% and 31.9% of the respective variances (Table 4).

**Table 4:** Multivariate regression analysis of factors associated with Scr/BUN levels in MHD patients.

DV	IV	RC	SE	*t*	P	*R*^2^
Scr	SDS	0.092	0.155	1.085	0.234	0.278
	SAS	0.198	0.067	4.637	0.000	
BUN	SDS	0.152	0.087	2.055	0.067	0.319
	SAS	0.269	0.209	5.233	0.000	

DV = Dependent Variable, IV = Independent Variable, RC = Regression Coefficient, SE = Standard Error.

## Discussion

Patients undergoing maintenance hemodialysis (MHD) face not only the physical burden of uremia and its complications but also considerable psychological distress, such as depression and anxiety, which further diminishes their quality of life^12,13^. Therefore, developing safe and engaging non-pharmacological interventions to improve psychological well-being and potentially affect solute concentrations is of clinical value.

Previous research has indicated that greater perceived benefits of exercise and fewer barriers to maintenance are associated with enhanced exercise regularity and functional capacity^14^. By comparing the depression and anxiety levels of the two groups before and after the intervention, it was found that the SDS and SAS scores of the study group were significantly lower than those of the control group. The intervention yielded a “large effect” on anxiety and depression reduction, indicating that VR pedal bike exercise care is beneficial in reducing negative emotions. Clinically, it can be combined with both to more efficiently improve patients’ mental health. The reason is that exercise is an important measure to improve the physical functions of MHD patients and reduce complications^15^. The VR exercise mode can stimulate patients’ exercise willingness and improve exercise effects. Rhee et al.^16^ studied that pedal bike exercise during the dialysis process can enhance patients’ self-confidence and independence, improve anxiety and depression and other psychological conditions.

The significant reduction in depression and anxiety scores observed in the VR group can be attributed to a synergistic effect. First, the physical exercise component itself is known to promote endorphin release. Second, and crucially, the immersive VR environment likely provided a powerful cognitive distraction from the monotonous and stressful dialysis procedure. This is supported by our prior research indicating that VR inherently enhances engagement and adherence to exercise protocols. By making the exercise session more enjoyable and tolerable, the VR component may have ensured sufficient dose and continuity of the exercise stimulus, which is essential for eliciting psychological benefits.

The changes in Scr and BUN levels and reduced clearance of inflammatory cytokines in MHD patients often result in a chronic micro-inflammatory state, which is closely associated with accelerated disease progression, increased complications, and elevated mortality^17^. Evidence suggests that physical exercise can mitigate inflammatory responses, enhance immune regulation, and contribute to the stabilization of Scr and BUN levels^18^. Serum creatinine (Scr), a metabolite of muscle metabolism, and blood urea nitrogen (BUN), the end product of protein metabolism, are key biomarkers reflecting renal impairment. In the present study, both groups exhibited a decline in Scr and BUN levels following the intervention, with a significantly more pronounced reduction observed in the VR exercise group. The intervention yielded a “large effect” on Scr/BUN reduction, suggesting that VR-cycling is not only statistically superior but also constitutes a “clinically meaningful” non-pharmacological therapy for alleviating Scr/BUN in MHD patients. Noguchi et al.^19^ demonstrated that cycling during hemodialysis significantly enhances phosphate removal and improves dialysis adequacy. It is critical to note that pre-dialysis BUN and Scr are influenced by numerous factors and are not direct measures of dialysis adequacy.

Our analysis further revealed a positive correlation between Scr/BUN levels an SDS/SAS scores. Regression analyses indicated that anxiety levels were significant predictors of Scr and BUN. This suggests that improvements in psychological well-being following VR-based bicycle exercise are associated with better renal outcomes. The findings of this study indicate that anxiety scale scores in MHD patients are not only significantly positively correlated with Scr and BUN levels but also serve as a significant predictor for their variation. A study involving over 340,000 participants from the UK Biobank revealed a clear dose–response relationship between the severity of cardiovascular-kidney-metabolic (CKM) syndrome and the risk of anxiety, suggesting a profound pathophysiological interplay between cardiorenal metabolic health and mental status^20^. The underlying mechanisms are likely to involve shared pathways mediated by chronic inflammation. Anxiety can promote the release of pro-inflammatory cytokines, and a state of chronic inflammation not only forms a biological basis for anxiety but can also directly change Scr and BUN levels^21^. Furthermore, the overactivation of the hypothalamic–pituitary–adrenal (HPA) axis and consequent autonomic nervous system dysregulation may also play a significant role in how anxiety exacerbates the burden on the kidneys^22^. It is noteworthy that this association is likely bidirectional; that is, deteriorating Scr and BUN levels can, in turn, exacerbate anxiety symptoms, creating a vicious cycle^23^. Consequently, integrating anxiety assessment into the routine management of MHD patients could serve as an early risk warning tool. Implementing non-pharmacological strategies, such as VR-based exercise interventions, which may alleviate anxiety and improve inflammatory status, offers a promising approach to breaking this cycle and improving patients’ long-term prognosis.

Our preliminary work has demonstrated that VR technology can significantly enhance exercise adherence in similar clinical populations by increasing motivation and reducing perceived exertion. Howerver, this study still has several limitations. First, the use of self-reported scales may be subject to bias. Second, the sample size was relatively small, and the age range (18–55 years) may limit the generalizability of findings to the typically older hemodialysis population. Third, Scr and blood BUN are influenced by numerous factors beyond dialysis clearance, including nutrition, muscle mass, and residual renal function. Their decrease in this study should not be interpreted as a direct measure of improved renal function or dialysis adequacy. In future studies, we will incorporate standard measures of dialysis adequacy and solute clearance, such as single-pool Kt/V (spKt/V), urea reduction ratio (URR), and serum levels of phosphate and β2-microglobulin. These direct metrics will provide more robust and clinically relevant evidence regarding the intervention’s impact on dialysis efficacy.

### Limitations and future directions

In summary, the application of VR-based bicycle exercise during MHD not only reduces negative emotional symptoms but also enables patients to achieve improved Scr and BUN levels in an engaging and low-stress environment. These findings support the clinical integration of VR-based exercise protocols into standard hemodialysis care.

## Data Availability

The datasets generated during the current study are not publicly available due to concerns regarding patient privacy and the conditions of ethical approval and informed consent. The data contain sensitive clinical and psychological information of a vulnerable patient population, and public deposition would compromise participant confidentiality. However, de-identified data are available from the corresponding author on reasonable request, subject to approval from the institutional ethics committee.

## Declarations

### Clinical trial registration number

Clinical trial registration number is ChiCTR-OOC-16007994. Date of registration: 2016/02/25.
